# Peritumoral/vascular expression of PSMA as a diagnostic marker in hepatic lesions

**DOI:** 10.1186/s13000-020-00982-4

**Published:** 2020-07-23

**Authors:** Wei Chen, Zhenghong Lee, Amad Awadallah, Lan Zhou, Wei Xin

**Affiliations:** 1 Department of Pathology, University Hospital Cleveland Medical Center, Case Western Reserve University, 10900 Euclid Ave, Cleveland, Ohio 44106 USA; 2 Department of Radiology, University Hospital Cleveland Medical Center, Case Western Reserve University, 10900 Euclid Ave, Cleveland, Ohio 44106 USA

**Keywords:** PSMA, Cholangiocarcinoma, Metastatic pancreatic ductal adenocarcinoma, Peritumoral expression, Diagnostic marker

## Abstract

**Background:**

The differential diagnosis between primary cholangiocarcinoma and metastatic pancreatobiliary adenocarcinoma is histologically challenging due to lack of distinct morphological features and reliable molecular markers. Prostate-specific membrane antigen (PSMA) is expressed in prostate epithelium and upregulated on the surface of prostatic adenocarcinoma cells. Studies have shown PSMA enzymatic activity is involved in malignancy-driven neoangiogenesis in the endothelium of tumor-associated neovasculature in breast, lung, thyroid, hepatocellular carcinoma (HCC) and urothelial cancer. Recently, PSMA-targeted imaging technology (PSMA PET-CT) detected the presence of PSMA in primary cholangiocarcinoma. However histological correlation with PSMA expression other mass lesions in the liver has not yet been studied.

**Methods:**

72 cases of liver mass resection were collected at a tertiary hospital from 2011 to 2019. Immunohistochemical stains for PSMA and CD34 were performed. The expression of PSMA in tumor cells and associated neovascular endothelium were analyzed separately and the locations of vascular structures were confirmed by CD34 expression.

**Results:**

Among 72 cases, 28 cases (22/72, 38.9%) showed PSMA peritumoral/vascular expression only, 3 cases (3/72, 4.2%) showed tumor cell expression only, and 2 cases (2/72, 2.8%) showed both tumor cell and peritumoral/vascular expression. The remainder (39/72, 54.2%) showed no expression. Particularly, most of primary cholangiocarcinoma showed PSMA vascular expression (13/15, 86.7%), while none of the 18 cases of metastatic pancreatobiliary adenocarcinoma were positive for PSMA (0/18, 0%) (*p* < 0.01). Outside of pancreatobiliary adenocarcinoma, none of the metastatic tumors, including colon and lung cancers, expressed PSMA. In 8 cases of metastatic prostate carcinoma, 3 showed PSMA expressions in tumor cells only (3/8, 37.5%) and 2 expressed PMSA in both tumor cells and neovasculature (2/8, 25.0%). Out of 22 HCC cases, 15 (15/22, 68.2%) were positive for PSMA in tumor vasculature. None of the 5 hepatic adenoma expressed PSMA (0/5, 0%).

**Conclusion:**

Significantly enhanced tumor-associated neovascular PSMA expression was identified in primary cholangiocarcinoma, compared to metastatic pancreatobiliary adenocarcinoma. Our findings potentially provide a sensitive marker in differential diagnosis between otherwise morphologically indistinguishable cases.

## Background

Cholangiocarcinomas arise from the epithelial cells of the bile ducts. Although rare in US, these cancers are highly lethal because most are locally advanced at the time of initial diagnosis. According to the SEER database, approximately 8000 new cases were reported each year in US [1]. Despite multidisciplinary interventions, including surgery, chemotherapy and radiotherapy, there is limited improvement in survival. Only 10% of patients with extrahepatic cholangiocarcinoma and 8% of patients with intrahepatic cholangiocarcinoma survive for 5 years or longer [2]. Additionally, the actual number of cases is likely to be higher because these cancers can be hard to diagnose and may be misclassified. Intrahepatic cholangiocarcinoma considered a primary liver tumor, shares histological and immunohistochemical features with many other primary and metastatic tumors, especially pancreatic ductal adenocarcinoma. The differential diagnosis between primary cholangiocarcinoma and metastatic pancreatic ductal adenocarcinoma is extremely important for clinical management but there are no reliable molecular markers to differentiate these 2 morphological similar entities.

PSMA is a 100 kDa type II-transmembrane glycoprotein with folate hydrolase and neurocarboxypeptidase activity and is expressed in prostate epithelium and upregulated on the surface of prostatic adenocarcinoma cells [3]. PSMA has diagnostic and therapeutic importance and PSMA-based imaging technology (PSMA PET-CT) and targeted therapy have been used for prostate cancer management [4, 5]. Studies have shown that PSMA enzymatic activity is involved malignancy-driven neoangiogenesis and is expressed in the endothelium of tumor-associated neovasculature of breast, lung, thyroid, hepatocellular carcinoma (HCC), transitional cell carcinoma of the urinary bladder, and a small portion of prostate adenocarcinoma [6, 7]. However, these studies focused on malignancy at the primary site and none of them explored the metastasis loci in liver.

Recently, the presence of PSMA in primary cholangiocarcinoma was detected by PSMA PET-CT [8]. However, no similar findings were reported for metastatic pancreatic ductal adenocarcinoma in liver. Our study evaluates the histology and imaging correlation of PSMA for intrahepatic cholangiocarcinoma and metastatic pancreatic ductal adenocarcinoma. We also investigated PSMA expression in other hepatic masses that are commonly seen in daily practice, including primary and metastatic lesions that may potentially interfere with cholangiocarcinoma diagnosis. We speculated that PSMA expression is different between primary cholangiocarcinoma and metastatic pancreatic ductal adenocarcinoma in the liver.

## Materials and methods

### Patients and Clinicopathologic data

This study was approved by the Institutional Review Board of University Hospitals Cleveland Medical Center. In total, 72 formalin-fixed, paraffin-embedded tissue blocks of liver mass resections from 72 patients were studied. Detailed clinicopathological data were retrieved from pathology reports/clinical records, including demographics, viral hepatitis status, ethanol history, and other possible cirrhosis-related causes.

### Assessment of PSMA expression

PSMA expression was evaluated by immunohistochemistry stain (IHC), which was performed by the Immunohistochemistry Diagnostic Laboratory of University Hospitals Cleveland Medical Center. Tissue slides were processed using a BenchMark Ultra automated immunostainer (Ventana, Tucson, AZ). Slides were de-paraffinized, antigen retrieved with standard Cell Conditioning 1 (Ventana Medical Systems, AZ), placed in a tris-based buffer pH 8.3 solution for 64 min at 95 C, then incubated at 37 C with the primary antibody PSMA mouse monoclonal antibody (1:200 dilution, Catalog #NCL-L-PSMA, clone 1D6 from Leica Biosystems, Newcastle, United Kingdom) for 24 min.

**Fig. 1:**
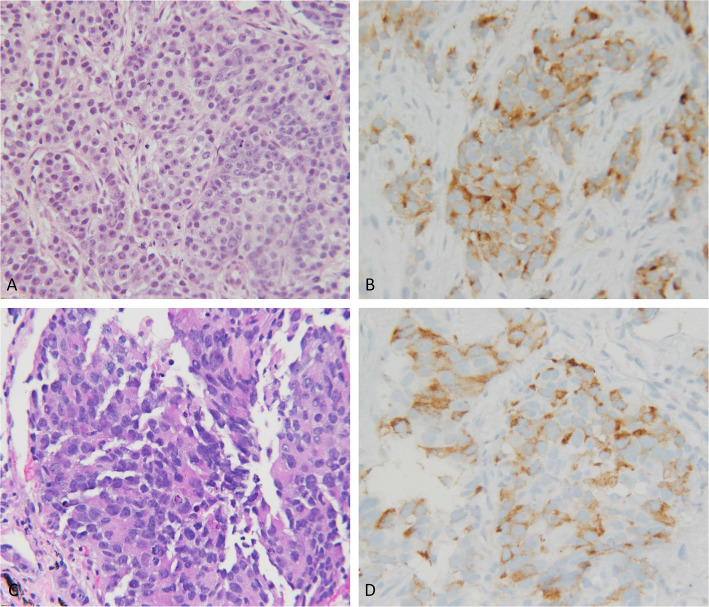
Prostate adenocarcinoma showed PSMA positivity in tumor cell pattern. **a**. Primary prostate adenocarcinoma (PSMA, 200x); **b**. Metastatic prostate adenocarcinoma (PSMA, 200x); **c**, **d**. Metastatic prostate adenocarcinoma (C. HE, 200x; D. PSMA, 200x)

**Fig. 2:**
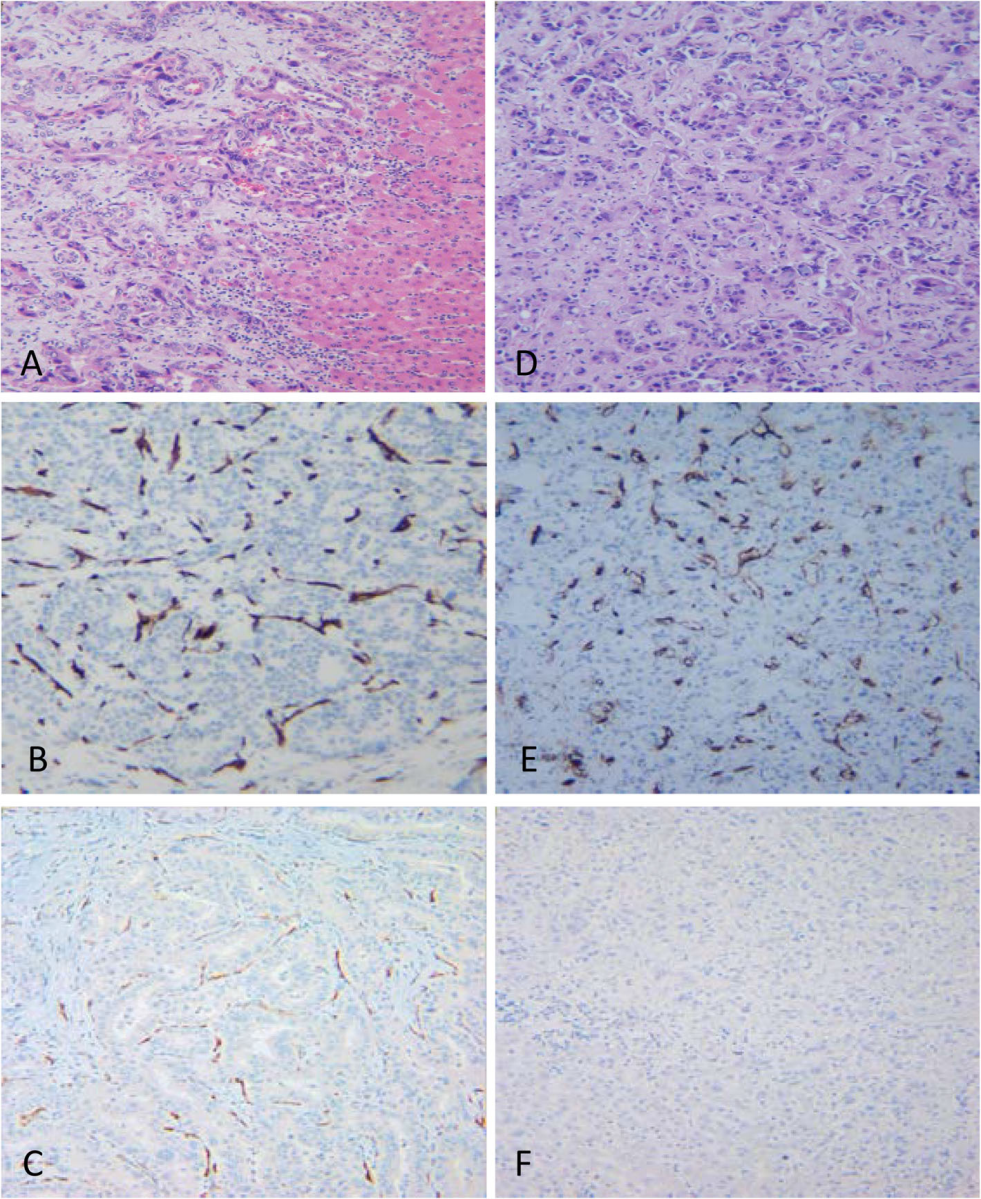
Primary cholangiocarcinoma and metastatic pancreatic ductal adenocarcinoma. **a**. Primary cholangiocarcinoma (HE, 100x); **b**. CD34 expression in cholangiocarcinoma (CD34, 200x); **c**. PSMA peritumoral/vascular expression in cholangiocarcinoma (PSMA, 200x). **d**. Metastatic pancreatic ductal adenocarcinoma in liver (HE, 100x) **e**. CD34 expression in pancreatic ductal adenocarcinoma (CD34, 200x); **f**. PSMA is negative in metastatic pancreatic ductal adenocarcinoma (PSMA, 200x)

PSMA expression was evaluated independently by two pathologists (WX and WC) on immunostained sections. Evaluation was blind with respect to clinical data. Any PSMA reactivity in either tumor cells or neoplastic vessels was considered positive (Figs. 1 and 2).

### Assessment of CD34 expression

The identity of vascular structures was confirmed by CD34 expression with IHC (Leica, Newcastle, UK). Unstained 4 μm-sections were prepared from paraffin blocks and baked for 30 min at 60 °C in a Boekel Lab oven. The slides were then processed using a BondMax Automated Immunostainer. The slides were deparaffinized, antigen retrieved, incubated in primary antibody and subsequently counterstained onboard the automated instrument.

### Statistical analysis

Statistical analyses were performed with SPSS 16.0 (SPSS Inc., Chicago, IL). Correlations between categorical variables were assessed using Chi-Square/Fisher’s Test. Bilateral logistic regression analyses were performed to evaluate the association between PSMA expression and clinicopathologic characteristics in HCC patients, including liver cirrhosis, hepatic viral status and alcohol history. We considered a *p* value less than 0.05 statistically significant.

## Results

### Patients

A total of 72 patients who underwent liver mass resection between 01/01/2011 and 12/31/2019 at the University Hospitals Cleveland Medical Center were enrolled in this study, with a mean age of 63.2 years (range of 29 to 88 years) and a male-to-female ratio of 2.2:1. The ethnic distribution is Caucasian 44 (61.1%), African American 15 (20.8%), Asian 4 (5.6%) and others/unknown 9 (12.5%).

Among the 72 cases, 42 were primary tumors and 30 were metastatic tumors (Table 1). HCC accounted for more than half of all the tumors in primary group (22/42, 52.4%); Cholangiocarcinoma accounted for approximately one third (15/42, 35.7%). Additionally, there were 5 cases of benign hepatic adenoma (5/42, 11.9%). For the 30 metastatic tumors examined, we found most originated from the pancreas (18/30, 60%) and prostate (8/30, 26.7%). The remaining tumors originated from the colon (3/30, 10.0%) and lung (1/30, 3.3%).

**Table 1:** PSMA vascular expression in primary and metastatic tumors

	PSMA vascular expression		
	Case (n)	Positive (n,%)	Negative (n,%)
Primary	42	28(66.7%)	14(33.3%)
HCC	22	15(68.2%)	7(31.8%)
Cholangiocarcinoma	15	13(86.7%)	2(13.3%)
Hepatic adenoma	5	0(0.0%)	5(100.0%)
Metastatic	30	2(6.7%)	28(93.3%)
Pancreatic ductal	18	0(0.0%)	18(100.0%)
Other sites*	12	2(16.6%)**	10(83.3%)

*Other sites includes liver metastatic tumor original from colon (3), lung (1) and prostate (8)

**In total 5 cases of metastatic prostate cancer showed positivity in PSMA, 2 of which had both tumor cell and peritumoral/vascular pattern, and the rest 3 cases were tumor cell positive only (counted in negative column)

### PSMA and CD34 expressions patterns

Three patterns of PSMA expression were identified: tumor cell, peritumoral/vascular, and no expression. Tumor cell expression appeared as a membrane stain of tumor cells. Staining was evenly distributed in the tumor area without peripheral/central alternation. (Fig. 1a-d) Vascular/peritumoral expression was identified as endothelial stains of the vessel, which was confirmed by CD34 positivity and tended to be more prominent at the tumor interface. (Fig. 2a, c) No expression was defined by a lack of PSMA expression in both the tumor and vascular structure. (Fig. 2d, f) In all cases, CD34 immunostains were performed to highlight the vessel presence and density. (Fig. 2b, e) Among 72 cases, 28 cases (28/72, 38.9%) showed only PSMA peritumoral/vascular expressions only, 3 cases (3/72, 4.2%) showed tumor cell expressions only, and 2 cases (2/72, 2.8%) showed both tumor cell and peritumoral/vascular expression. The remaining cases (39/72, 54.2%) had no expression.

PSMA expression in cholangiocarcinoma and other metastatic adenocarcinomas.

Most primary cholangiocarcinoma showed PSMA peritumoral/vascular expression (13/15, 86.7%). In contrast, none of the 18 cases of metastatic pancreatic ductal adenocarcinomas were positive for PSMA (0/8, 0.0%) (*p* < 0.01) (Table 1). Additionally, none of the colon and lung metastatic tumors expressed PSMA. Metastatic prostate carcinomas, however, were PSMA positive in 5 out of 8 cases (62.5%). Of the 5 PSMA positive cases, only 2 cases had both tumor cell and peritumoral/vascular expression pattern. In all cases, CD34 stains confirmed the presence of vessels.

### PSMA expression in HCC and hepatic adenoma

In primary tumors, none of the hepatic adenoma demonstrated expressions (0/5, 0.0%). Of the 22 cases of HCC, 15 had peritumoral/vasculature expression (15/22, 68.2%). No tumor cell pattern positivity was identified. Among HCC, 6 cases were grade 3 (27.3%), 15 cases were grade 2 (68.2, and none were grade 1. One case was fibrolamellar HCC (4.5%). PSMA peritumoral/vascular expression was more likely to be identified in grade 3 HCC (5/6, 83.3%) but was also found in grade 2 cases (10/15, 66.7%). PSMA was negative in the case with fibrolamellar HCC. PSMA was not expressed in any normal liver tissue or in non-neoplastic cirrhosis.

Association of PSMA expression and clinicopathologic characteristics in HCC.

Clinicopathologic information was collected for enrolled patients, including viral hepatitis status, ethanol history, and other possible cirrhosis-related causes. Among the patients with HCCs, 10 had cirrhotic background and 12 did not. PMSA peritumoral/vascular expression was significantly more common in HCC with cirrhosis (9/10, 90.0%) than in non-cirrhotic HCC (6/12, 50%) (*p* < 0.05), see Table 2. Further analysis showed that in 15 PSMA positive cases, 11 (73.3%) had Hepatitis C or Hepatitis B infection, significantly higher than for the non-viral infected cohort (4/15, 26.7%) (*p* = 0.03).

**Table 2:** PSMA neovascular expression in primary liver tumors

	HCC		Adenoma
	Cirrhotic	Non-Cirrhotic	
PSMA			
Positive	9(90.0%)*	6(50.0%)	0(0.0%)
Negative	1(10.0%)	6(50.0%)	5(100.0%)

*p < 0.05, compared to non-cirrhotic HCCs

## Discussion

We showed novel evidence that significantly enhanced tumor-associated neovascular PSMA expression is present in primary cholangiocarcinoma but not in metastatic pancreatic ductal adenocarcinoma, although the two are morphologically indistinguishable. Our finding is consistent with recent report that primary cholangiocarcinoma could be visualized in liver with PSMA traced PET-CT scan [8]. Although the case number was relatively limited, this finding could provide a potential marker for differentiating diagnosis.

Tumor cell PSMA expression was mainly reported in both benign and malignant prostate tissue, while neovascular expression was found in a wider spectrum of malignant neoplasms [6, 7, 9, 10]. Chang et al. identified high frequency of neovascular expression in epithelial tumors (such as carcinomas, including renal cell carcinoma, transitional cell carcinoma, pancreatic ductal adenocarcinoma, breast carcinoma and colonic adenocarcinoma), neuroendocrine tumors, melanoma, and non-vascular soft tissue sarcomas [6]. In contrast to previous studies, we focused on metastasis loci instead of primary lesions. Metastatic carcinomas, such as pancreatic ductal and colonic adenocarcinoma, showed reduced PSMA neovascular expression compared to their primary sites.

The exact role of PSMA in tumor-associated neoangiogenesis is not fully understood. An animal model study showed that angiogenesis was severely impaired in PSMA-null mice. This angiogenic defect occurred at the level of endothelial cell invasion through the extracellular matrix barrier [11]. The author also revealed PSMA as a principal component of a regulatory loop that modulates laminin-specific integrin signaling and GTPase-dependent, p21-activated kinase 1 (PAK-1) activity [11]. On the other hand, tumor desmoplasia and hypoxia were also associated with neoangiogenesis [12, 13]. A more recent study showed that prominent stromal fibrosis was common in pancreatic ductal adenocarcinoma and played a role in regulation of angiogenesis [13]. Based on the above findings, we can propose two possible reasons for the different PSMA profiles between primary and metastatic sites. First, PMSA expression is related to differences in the severity of hypoxia between the primary and metastatic site. Generally, hypoxia is more common at the primary site and more severe with large tumors. In contrast, metastatic sites have more available blood supply and thus less risk of hypoxia stress. Therefore, hypoxia-induced angiogenesis would be less prominent in metastatic site, and PSMA, as a part of neoangiogenesis, would be negatively regulated. Second, desmoplasia appears more frequently in primary sites compared to metastatic loci, at least in the case of pancreatic ductal adenocarcinoma. Therefore, desmoplasia-related neoangiogenesis, as well as PSMA, would be down-regulated in metastatic lesions, leading to PSMA loss in metastatic malignant tissues.

To better encompass the scope of lesions that are frequently seen in liver, we also explored PSMA expression in HCC, the most common primary malignancy in liver. In our study showed, 68% of the cases of HCC had neovascular PSMA expression, consistent with previous studies. However, the positive rates among previous reports showed disagreement [7, 10, 14]. Tolkach et al. reported more than 80% of tumors showed vascular expression in 158 cases of HCC [10]. However, in another analysis, the author found weak PSMA expression in only 3 out of 44 HCC cases (6.8%) [7]. The inconsistent observation might reflect the heterogeneous nature in PSMA expression in HCCs. In fact, we found that PSMA peritumoral/vascular expression was significantly higher in tumors arising from cirrhotic background with hepatic virus infection. Per our data, 90% of cases of HCC with cirrhosis were PMSA peritumoral/vascular positive, much higher than expression in non-cirrhotic HCC (6/12, 50%). Further analysis showed 73.3% PSMA positive cases had Hepatitis C or Hepatitis B infection, significantly higher than the non-viral infected cohort. Since previous studies did not provide cirrhotic information, a definitive conclusion cannot yet be made. Regardless, our findings shed light on the role of PSMA as an important ancillary marker to help differentiate HCCs from cirrhotic nodules in morphologically challenging cases, since cirrhotic liver background has shown to be consistently negative for PSMA peritumoral/vascular expression in our study and many previous reports [15–18]. Other clinicopathological factors that might interfere PSMA expression included tumor differentiation. Recently, Jiao et al. reported that in 103 cases of HCC cohort, patients with moderate/poorly differentiated tumors were more likely to have high PSMA expression, compared to patients with well-differentiated tumors. Patients with high tumor stage and lymph node metastasis tended to have high PSMA expression [14]. Our study also found a similar trend, as more than 80% patients with Grade 3 HCCs had PMSA peritumoral/vascular expression compared to only two-thirds of patients with Grade 2 HCCs. However, no statistical significance was reached due to limited sample volume. Additionally, the mechanism behind higher frequency of PSMA expression with cirrhotic background is not well characterized yet. Whether PSMA played a role in poorly differentiated HCC carcinogenesis needs further investigation as well.

To the best of our knowledge, we are the first group to report lack of PMSA expression in hepatic adenoma regardless of subtypes. Based on the known mechanism of PSMA in neoangiogenesis, our finding was expected and consistent with results from other benign tumors. Mhawech-Fauceglia et al. showed PSMA was negative in all 846 benign tumors they tested, which included neurofibroma, lipoma, leiomyoma, colon polyps, thyroid adenoma and others [7].

We only noticed PMSA expression in tumor cells of metastatic prostate cancer. Additionally, it was not surprising that only 62.5% tumors were PMSA positive in our study as a very similar expression rate was reported previously in a larger cohort [7]. Overall, 93 out of 141 (66.0%) prostate adenocarcinoma cases were cellularly positive for PSMA. Treatment history and hormonal secretion status may affect the expression of PSMA. However, we also found a low rate of peritumoral/vascular expression in metastatic prostate cancer. Morphological and clinical correlation were need in diagnosis.

In summary, our study profiled PSMA expression in common hepatic masses. We identified significantly enhanced tumor-associated neovascular PSMA expression in primary cholangiocarcinoma and not identified in metastatic pancreatic ductal adenocarcinoma. We also showed higher frequency of neovascular PSMA expression in HCCs arising from cirrhotic liver background and provided a potential marker for differentiating tumor nodules from cirrhotic nodules in difficult cases. Although the mechanism was not fully uncovered, the unique role of PSMA in tumor neoangiogenesis might extend the diagnostic and therapeutic value of PMSA analysis from prostate cancer to more solid malignancies.

## Data Availability

The datasets used and/or analyzed during the current study are available from the corresponding author on reasonable request.

## Abbreviations

PSMA: Prostate-specific membrane antigen
IHC: Immunohistochemistry stain
HCC: Hepatocellular carcinoma

## References

[1] Howlader N, Noone A, Krapcho M, Miller D, Brest A, Yu M, et al. SEER Cancer Statistics Review, 1975–2016 Bethesda, MD: National Cancer Institute; 2019 [https://seer.cancer.gov/csr/1975_2016/, based on November 2018 SEER data submission, posted to the SEER web site, April 2019.] Last acessed: 12/2019.

[2] Antwi SO, Mousa OY, Patel T (2018). Racial, ethnic, and age disparities in incidence and survival of intrahepatic Cholangiocarcinoma in the United States; 1995-2014. Ann Hepatol.

[3] Pinto JT, Suffoletto BP, Berzin TM, Qiao CH, Lin S, Tong WP (1996). Prostate-specific membrane antigen: a novel folate hydrolase in human prostatic carcinoma cells. Clin Cancer Res.

[4] Evangelista L, Briganti A, Fanti S, Joniau S, Reske S, Schiavina R (2016). New clinical indications for (18)F/(11)C-choline, new tracers for positron emission tomography and a promising hybrid device for prostate Cancer staging: a systematic review of the literature. Eur Urol.

[5] Kratochwil C, Giesel FL, Stefanova M, Benešová M, Bronzel M, Afshar-Oromieh A (2016). PSMA-targeted radionuclide therapy of metastatic castration-resistant prostate Cancer with 177Lu-labeled PSMA-617. J Nucl Med.

[6] Chang SS, Reuter VE, Heston WD, Bander NH, Grauer LS, Gaudin PB (1999). Five different anti-prostate-specific membrane antigen (PSMA) antibodies confirm PSMA expression in tumor-associated neovasculature. Cancer Res.

[7] Mhawech-Fauceglia P, Zhang S, Terracciano L, Sauter G, Chadhuri A, Herrmann FR (2007). Prostate-specific membrane antigen (PSMA) protein expression in normal and neoplastic tissues and its sensitivity and specificity in prostate adenocarcinoma: an immunohistochemical study using mutiple tumour tissue microarray technique. Histopathology..

[8] Marafi F, Usmani S, Esmail A (2019). 68Ga-prostate-specific membrane antigen PET/CT in Cholangiocarcinoma: a potential biomarker for targeted Radioligand therapy?. Clin Nucl Med.

[9] Kinoshita Y, Kuratsukuri K, Landas S, Imaida K, Rovito PM, Wang CY (2006). Expression of prostate-specific membrane antigen in normal and malignant human tissues. World J Surg.

[10] Tolkach Y, Goltz D, Kremer A, Ahmadzadehfar H, Bergheim D, Essler M (2019). Prostate-specific membrane antigen expression in hepatocellular carcinoma: potential use for prognosis and diagnostic imaging. Oncotarget..

[11] Conway RE, Petrovic N, Li Z, Heston W, Wu D, Shapiro LH (2006). Prostate-specific membrane antigen regulates angiogenesis by modulating integrin signal transduction. Mol Cell Biol.

[12] Azoitei N, Becher A, Steinestel K, Rouhi A, Diepold K, Genze F (2016). PKM2 promotes tumor angiogenesis by regulating HIF-1α through NF-κB activation. Mol Cancer.

[13] Lunardi S, Muschel RJ, Brunner TB (2014). The stromal compartments in pancreatic cancer: are there any therapeutic targets?. Cancer Lett.

[14] Jiao D, Li Y, Yang F, Han D, Wu J, Shi S (2019). Expression of prostate-specific membrane antigen in tumor-associated vasculature predicts poor prognosis in hepatocellular carcinoma. Clin Transl Gastroenterol.

[15] Haffner MC, Kronberger IE, Ross JS, Sheehan CE, Zitt M, Mühlmann G (2009). Prostate-specific membrane antigen expression in the neovasculature of gastric and colorectal cancers. Hum Pathol.

[16] Silver DA, Pellicer I, Fair WR, Heston WD, Cordon-Cardo C (1997). Prostate-specific membrane antigen expression in normal and malignant human tissues. Clin Cancer Res.

[17] Liu H, Moy P, Kim S, Xia Y, Rajasekaran A, Navarro V (1997). Monoclonal antibodies to the extracellular domain of prostate-specific membrane antigen also react with tumor vascular endothelium. Cancer Res.

[18] Mahalingam D, Peguero J, Cen P, Arora SP, Sarantopoulos J, Rowe J, et al. A Phase II, Multicenter, Single-Arm Study of Mipsagargin (G-202) as a Second-Line Therapy Following Sorafenib for Adult Patients with Progressive Advanced Hepatocellular Carcinoma. Cancers (Basel). 2019;11(6). 10.3390/cancers11060833 PMC6627768 31212948

